# Blocking lactate: kick T cells when they are down

**DOI:** 10.1097/IN9.0000000000000059

**Published:** 2025-03-19

**Authors:** Veronika Horkova, Bart Everts, Dirk Brenner

**Affiliations:** 1 Experimental and Molecular Immunology, Department of Infection and Immunity (DII), Luxembourg Institute of Health, Esch-sur-Alzette, Luxembourg; 2 Immunology & Genetics, Luxembourg Centre for Systems Biomedicine (LCSB), University of Luxembourg, Esch-sur-Alzette, Luxembourg; 3 Leiden University Center for Infectious Diseases (LUCID), Leiden University Medical Center, Leiden, Netherlands; 4 Odense Research Center for Anaphylaxis (ORCA), Department of Dermatology and Allergy Center, Odense University Hospital, University of Southern Denmark, Odense, Denmark

**Keywords:** T cell exhaustion, MCT11, lactate, hypoxia, metabolism, immune checkpoint blockade, anti-tumor immunity

## Abstract

In the last couple of decades, cancer research has been shifting its focus to the immune system. Cancer cells, with their ability to adapt and evade immune responses, seem to accelerate the evolutionary pressure that has been put on our immune system during evolution. We thus try to aid these natural selection processes and assist our immune system to combat cancer. Here, we are discussing a study by Greg Delgoffe and colleagues that was published in *Nature Immunology* in December 2024, exploring a new approach to bring the dysfunctional immune cells back to life by blocking their lactate uptake.

The tumor microenvironment (TME) is a very harsh territory for immune cells to properly function. There are many mechanisms, which lead to suppression of the immune system in the TME ^[1,2]^. Continuous activation of T cells, together with negative signals derived from cancer cells, lead to dysfunction of T cells, more commonly known as “T-cell exhaustion” ^[3]^. This phenomenon is mainly described for CD8^+^ T cells, though CD4^+^ T cell exhaustion has been studied as well ^[4]^. Exhausted T cells are mostly characterized by upregulation of inhibitory coreceptors, such as PD-1, CTLA-4, TIM3, or LAG3. However, one has to bear in mind that these inhibitory molecules are primarily expressed on highly activated and functional T cells to provide a negative feedback loop that limits excessive activation and autoimmunity. Metabolic shifts in exhausted T cells directed by mitochondrial dysfunction lead to a decrease in cytokine production and proliferation ^[5]^. Decreased functionality is key for exhausted T cells and is regulated in distinct stages. The transition from functional cells, so-called “progenitor exhausted T cells” (T_PEX_) to “terminally exhausted T cells” (T_EX_) has been described to be defined mainly by a loss of transcription factor T cell factor 1 (TCF1). TCF1 drives a stem cell-like transcription program in these cells, enabling self-renewal ^[6]^. Thus, T_PEX_ in contrast to T_Ex_ resembles a partially functional state and still responds to immune checkpoint blockade (ICB) ^[7]^.

Only in the last couple of decades, the functionality of immune cells has been shown to be dependent on metabolic rewiring ^[8,9]^. In contrast, in cancer cells, a metabolic switch toward glycolysis and thus lactate overproduction was already described a century ago by Otto Warburg ^[10]^. Lactate efflux from highly proliferating cancer cells acidifies the TME and in parallel affects a variety of immune cells, including T cells. This is one mechanism that contributes to a more immune-suppressive environment in the TME ^[2,11]^. Lactate seems to also feed the hyper-proliferation of tumor cells by modulating their cell cycle ^[12]^. More recently, modification of histones directly by lactate has been described to alter mainly the expression of genes that are involved in the metabolic control of T cells ^[13]^. Lactate therefore seems to regulate a multitude of aspects of the antitumor response. The transport of lactate across the cell membrane is facilitated by monocarboxylate transporters (MCT) ^[14]^. Out of 16 members of this family of proton-linked transporters, MCT1–4 have been most extensively studied. Blockage of lactate efflux from cancer cells appears to be a very promising strategy to slow down cancer progression. In patients, higher expression of MCT1 and MCT4 is correlated with worse prognosis for multiple cancer types ^[15]^ and Phase I clinical trials with MCT1 inhibitors have demonstrated safety for their use in humans ^[16,17]^.

ICB therapy initiated a paradigm shift in cancer therapy by boosting the anticancer T cell response, rather than targeting the cancer cells themselves. However, because T_EX_ cells do not respond to ICB, they have been mostly considered as unusable in terms of antitumor responses. Peralta et al ^[18]^ now describe a surprising and promising strategy to reinvigorate the function of T_EX_ cells. Instead of targeting lactate efflux in tumor cells, the authors developed a strategy to block lactate import of T cells, thus limiting its detrimental effects ^[18]^.

Peralta et al ^[18]^ discovered that *Slc16a11* expression, which encodes the lactate transporter MCT11, was specifically upregulated in tumor-infiltrating murine and human T_EX_ cells. Using carbon tracing of lactate, the authors showed T_EX_ cells re-directed the influx of lactate-derived carbons to the tricarboxylic acid cycle (TCA) to generate CO_2_ compared to T_PEX_ and naïve CD8^+^ T cells ^[18]^. The authors further developed a conditional *Slc16a11* deletion in T cells, using an inversion model where, upon Cre recombination, *the Slc16a11* coding sequence was cut out and successful recombination led to green fluorescent protein (GFP) expression. This model allowed the authors to easily trace those T cells that at some point expressed *Slc16a11*. Peralta et al ^[18]^ showed that only about 39% of T_EX_ cells expressed *Slc16a11* and that even a subpopulation of intermediate T_EX_ (PD1^HIGH^TIm3^−^) showed *Slc16a11* expression ^[18]^. *Slc16a11*-deficiency abrogated the increase of lactate utilization within T_EX_ cells and restored lactate metabolism back to the level observed in naïve/T_PEX_ cells. The authors further showed that T_EX_ cells during chronic viral infections expressed higher levels of MCT11 and were more hypoxic. In line, repeated T-cell receptor (TCR)-stimulation of both murine and human CD8^+^ T cells in vitro led to higher MCT11 expression paired with a pronounced increase in intracellular hypoxia. As authors suggested hypoxia to be a major factor that determines MCT11 expression in T_EX_ cells, they explored the possibility of whether its expression is dependent on transcription factor Hif1α. However, Hif1α seemed to affect MCT11 expression only partially.

T cell-specific MCT11-deficient mice showed a higher infiltration of CD8^+^ T cells in tumors. However, the portion of T_EX_ cells did not seem to be altered, though the expression of co-inhibitory receptor TIM3 seemed to be decreased. Most importantly, MCT11 deficiency or its blockade in combination with anti-PD-1 treatment led to a doubling of the cytokine production of T_EX_ cells, indicating a gain of function. Further analysis showed reduced hypoxia in MCT11-deficient T_EX_ cells altering their transcriptomic program more towards the 1 characteristic for T_PEX_-like cells. Although the data point toward increased functionality, in murine melanoma, MCT11 deficiency itself did not affect the antitumor response. Only combination with ICB led to a slight boost of antitumor response further indicating that these cells acquire a T_PEX_-like state.

With promising data from the genetic model, Peralta et al ^[18]^ moved towards a more translational approach: treatment with MCT11-blocking antibodies ^[18]^. They show that tumor-infiltrating T_EX_ cells decrease lactate uptake upon MCT11 blockage. Moreover, the usage of MCT11-blocking antibodies in murine tumor models led to a moderate reduction in tumor burden. The therapeutic effect was especially strong in the murine head and neck squamous cell carcinoma (HNSCC) model, hinting at a possible monotherapeutic effect. In less responsive murine adenocarcinoma model, MCT11-blockade in combination with ICB almost doubled the efficacy of the therapy. The authors further showed that usage of MCT11-blockade together with chimeric antigen receptor (CAR) T cell adoptive therapy slightly improved the antitumor efficiency as well. Peralta et al ^[18]^ explored the functional changes in the tumor-infiltrating T_EX_ cells. The production of tumor necrosis factor (TNF) and interferon gamma (IFNγ) in T_EX_ cells upon MCT11-blockade did not improve in the poorly responding melanoma model ^[18]^. In contrast, in the HNSSC model, there was a significant increase in TNF and IFNγ production by tumor-infiltrating T_EX_ cells (Figure 1). The MCT11-blocking antibodies also led to less hypoxic tumor-infiltrating T_EX_ cells in the murine model of HNSCC. Interestingly, the combination between MCT11-blockade and ICB therapy in adenocarcinoma only moderately improved the cytokine production of T_EX_ cells compared to ICB alone but greatly reduced the tumor burden and improved survival.

These intriguing findings by Peralta et al ^[18]^ could represent a significant advancement in anticancer therapy, particularly for tumors such as adenocarcinoma or HNSCC ^[18]^. However, some questions are still to be answered. Does the MCT11 blockage change the lactylation of histones in proximity to mitochondrial genes or other genes that regulate metabolism? T cell exhaustion can be driven by mitochondrial dysfunction. Enhancement of hypoxic state within the T_EX_ cells is likely to further exacerbate their mitochondrial dysfunction ^[19]^. Therefore, a detailed assessment of mitochondrial function is needed. Mitochondrial dysfunction in hypoxia leads to increased production of mitochondrial reactive oxygen species (ROS) within T cells by so far unknown mechanism ^[19]^. Dysregulated ROS within T cells is detrimental for T cell function ^[20,21]^. As a consequence, their mitochondria adopt a state closely resembling that found in T_EX_^[22,23]^. The authors themselves previously described that continuous T cell stimulation within the TME could be the cause of increased ROS production in T_EX_ cells, driving their dysfunction. They show that T_EX_ functionality can at least partially be rescued by buffering the ROS ^[5]^. To better understand the molecular mechanisms of functional rescue of T_EX_ cells, it would also be important to unveil how MCT11 deficiency/blockage affects the level of ROS in the T_EX_ cells.

What actually drives the T_EX_ cells to upregulate MCT11 production? A plethora of cancer types upregulate the expression of lactate transporters ^[15]^ and lactate dehydrogenase (LDH), and indeed, the prognosis of the patients worsens with higher LDH expression ^[24]^. What happens when cancer cells run out of glucose and other energy sources to generate enough energy for survival within the TME? Lactate indeed can be utilized as a source of energy, and the reduced nicotinamide adenine dinucleotide (NADH) generated during the transformation of lactate back to pyruvate can be regenerated by desaturation of polyunsaturated fatty acids ^[25]^. Therefore, another potential strategy is the use of LDH inhibition, which has already been explored to reduce lactate production by cancer cells ^[26]^. However, could inhibition of LDH also affect the function of T_EX_ cells? Could the MCT11 upregulation be the last attempt of T_EX_ cells to adapt to the lactate overabundance, to take it as a source of energy, to open up the chromatin, and to boost the expression of the mitochondrial genes? Or could it be an attempt to increase the efflux of lactate? From the data provided by Peralta et al ^[18]^ it seems, though, this strategy is not helpful but rather detrimental for T cells.

**Figure 1.**
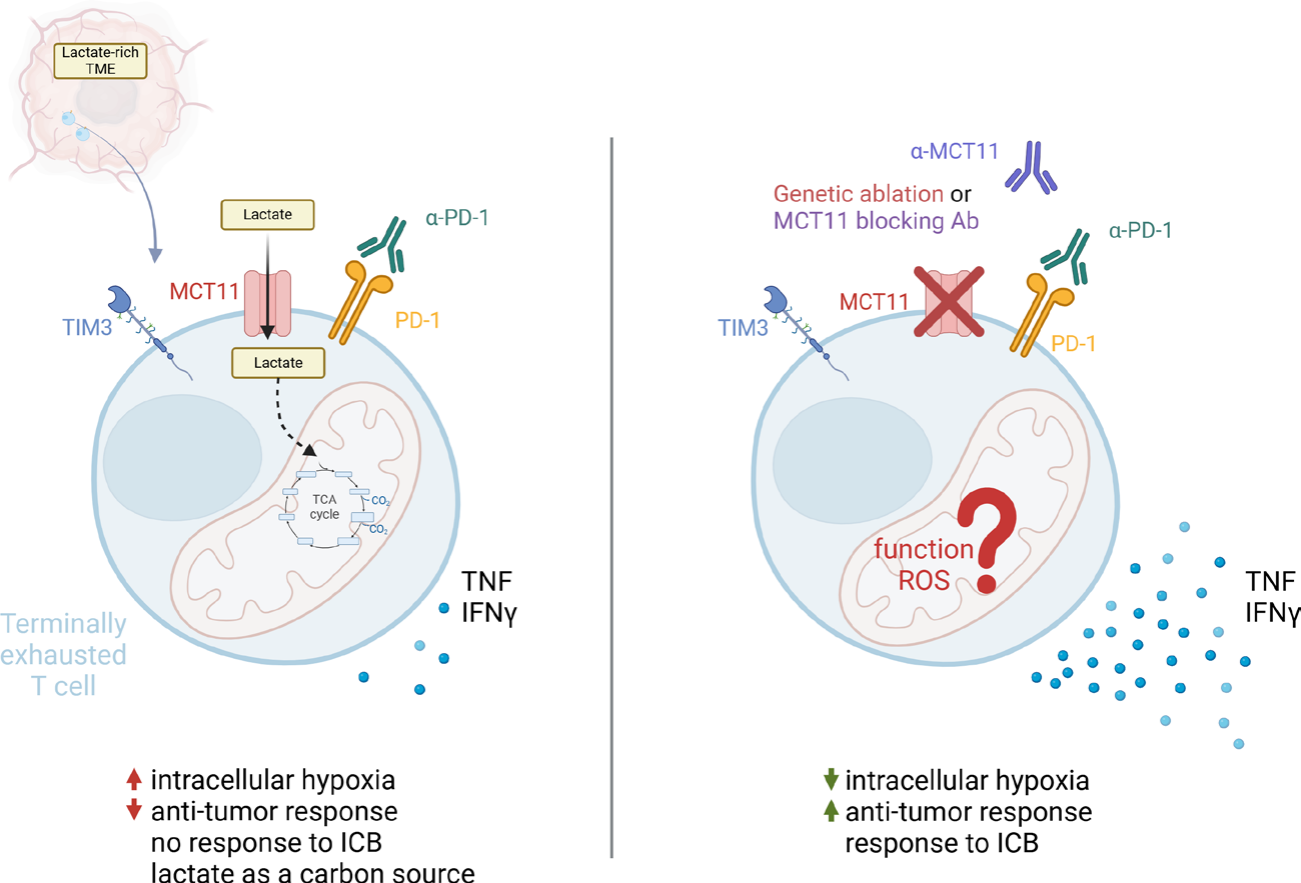
Terminally exhausted T cells (T_EX_) in a lactate-rich tumor environment import lactate via monocarboxylate transporter 11 (MCT11). The lactate influx leads to an increased population of hypoxic T_EX_ cells combined with reduced production of tumor necrosis factor (TNF) and interferon γ (IFNγ) in response to ICB (LEFT). Upon genetic ablation or utilization of antibody-mediated MCT11-blockade, the lactate influx to T_EX_ cell via MCT11 is diminished. This results in the reduction of intracellular hypoxia and better antitumor responses including increased TNF and IFNγ production in T_EX_ cells. MCT11 ablation significantly improves the T_EX_ response to ICB therapy (RIGHT). Created with BioRender.com.

## Conflicts of interest

The authors declare that they have no conflicts of interest.

## Acknowledgements

D.B. is supported by FNR-CORE grant C21/BM/15796788. V.H. is a recipient of an EMBO postdoctoral fellowship ALTF 138-2022. B.E. is supported by NWO VIDI grant 91719349.
